# Association between geriatric nutritional risk index and overactive bladder in the elderly population: a cross-sectional study

**DOI:** 10.3389/fnut.2025.1537549

**Published:** 2025-02-11

**Authors:** Wei Zheng, Chuanzan Zhou, Jia Miao, Yunkai Yang, Xuanhan Hu, Heng Wang, Xinyu Zhang, Qi Zhang, Yifan Wang

**Affiliations:** ^1^Emergency and Critical Care Center, Department of Emergency Medicine, Zhejiang Provincial People's Hospital, Hangzhou Medical College, Hangzhou, Zhejiang, China; ^2^Department of Nursing, Zhejiang Provincial People's Hospital, Hangzhou Medical College, Hangzhou, Zhejiang, China; ^3^Urology & Nephrology Center, Department of Urology, Zhejiang Provincial People's Hospital, Hangzhou Medical College, Hangzhou, Zhejiang, China; ^4^The Second Clinical Medical College, Zhejiang Chinese Medical University, Hangzhou, Zhejiang, China

**Keywords:** geriatric nutritional risk index, overactive bladder, elderly population, nutrition, NHANES database

## Abstract

**Background:**

The prevalence of overactive bladder (OAB) is increasing in the elderly population and there is growing evidence that malnutrition affects the urinary system. Despite this, research on the relationship between nutritional factors and OAB remains limited.

**Methods:**

We included 17,161 elderly individuals from the National Health and Nutrition Examination Survey conducted between 2005 and 2018. Overactive Bladder Symptom Scores (OABSS) were utilized to assess symptoms of OAB. A multifactorial logistic regression analysis was employed to evaluate the independent association between the Geriatric Nutritional Risk Index (GNRI) and the prevalence of OAB. Restricted cubic spline plots examined the potential non-linear relationship between GNRI and OAB. Influencing factors were assessed through subgroup analyses, while the predictive utility of GNRI was assessed with receiver operating characteristic (ROC) curves. The influence of inflammatory response and cognitive function on the interaction between GNRI and OAB was also examined by mediation analysis.

**Results:**

GNRI in the OAB group was significantly lower than that in the non-OAB group. Multifactorial logistic regression analysis revealed that GNRI significantly predicts OAB (*p* < 0.05). The Restricted Cubic Spline (RCS) curve indicated a non-linear negative correlation between GNRI and the risk of OAB in the elderly (non-linear *p* = 0.0029). In receiver operating characteristic analysis, GNRI outperforms serum albumin or body mass index (BMI) alone in predicting OAB risk. The study revealed that inflammatory response mediates the relationship between GNRI and OAB, while cognitive function has a relatively weaker influence on the strength of the association between GNRI and OAB.

**Conclusion:**

GNRI serves as a reliable predictive marker for OAB in the elderly population, demonstrating a nonlinear inverse correlation with OAB prevalence. Furthermore, this study elucidates the underlying inflammatory mechanisms that link GNRI to the development of OAB.

## Introduction

Overactive Bladder (OAB) is a prevalent urological condition characterized by urinary urgency, frequency, and nocturia in the absence of a discernible urinary tract infection or other pathological abnormalities (1). The prevalence of OAB among men in the United States is 16%, and for women, it is 16.9% (2, 3). Notably, the incidence of OAB increases significantly with age. OAB not only impacts the quality of life, sleep quality, and mental health of affected individuals but also imposes a substantial economic burden (4, 5). In the United States, the medical costs for patients with OAB are more than 2.5 times higher than for similar patients without OAB (6). Consequently, the management and treatment of OAB represent a significant challenge in the field of public health.

Nutritional deficiencies can have profound effects on the urinary system. Studies have indicated that there is an association between dietary nutrients and the occurrence of OAB, with higher intakes of protein and vitamin D being significantly correlated with a reduced risk of OAB development (7). Some potential mechanisms suggest that malnutrition may contribute to the development or exacerbation of OAB. Firstly, low serum albumin levels may lead to a decrease in vascular oncotic pressure, triggering compensatory diuretic mechanisms to maintain fluid balance. This could exacerbate OAB symptoms by increasing urinary frequency. Nutritional deficiencies may also impair muscle synthesis and maintenance, thereby altering the contractility and elasticity of the bladder wall (8). This can result in increased bladder irritability and reduced capacity, thereby triggering symptoms of OAB. Moreover, malnutrition may affect neurotransmitter signaling, which plays a crucial role in the regulation of micturition (9). Low serum albumin levels may lead to enhanced cholinergic signaling and subsequent detrusor overactivity (10).

The Geriatric Nutritional Risk Index (GNRI) is an indicator designed to evaluate the nutritional status of elderly individuals by combining serum albumin levels and body mass index to predict the risk of malnutrition (11). The GNRI is widely used in clinical and epidemiological studies due to its simplicity and ease of application (12, 13). Beyond assessing nutritional status, the GNRI has also been found to correlate with the prognosis of various diseases, such as heart failure, diabetes, and osteoporosis (14, 15). However, the relationship between GNRI and OAB has not been extensively studied, which limits our understanding of potential risk factors for OAB.

The National Health and Nutrition Examination Survey (NHANES) is a large-scale, population-based, cross-sectional survey that collects health and nutrition information on the civilian non-institutionalized population in the United States. The NHANES database, characterized by its extensive sample coverage and diverse array of indicators, offers comprehensive demographic, socioeconomic, dietary, health-related measurements, physiological assessments, laboratory tests, and additional information from across the United States. In this study, we leveraged the large-scale cross-sectional NHANES study to establish the correlation between GNRI and OAB in the elderly population, a finding that may hold broader implications for clinical practice. Identifying GNRI as a predictive factor for OAB can assist clinicians in prioritizing nutritional assessment and intervention measures for elderly patients at risk of developing OAB. Furthermore, by reducing the risk of OAB through nutritional interventions, healthcare providers may alleviate the economic burden associated with the treatment and management of OAB, thereby optimizing resource allocation and patient care.

## Methods

### Database and survey populations

**Figure 1:**
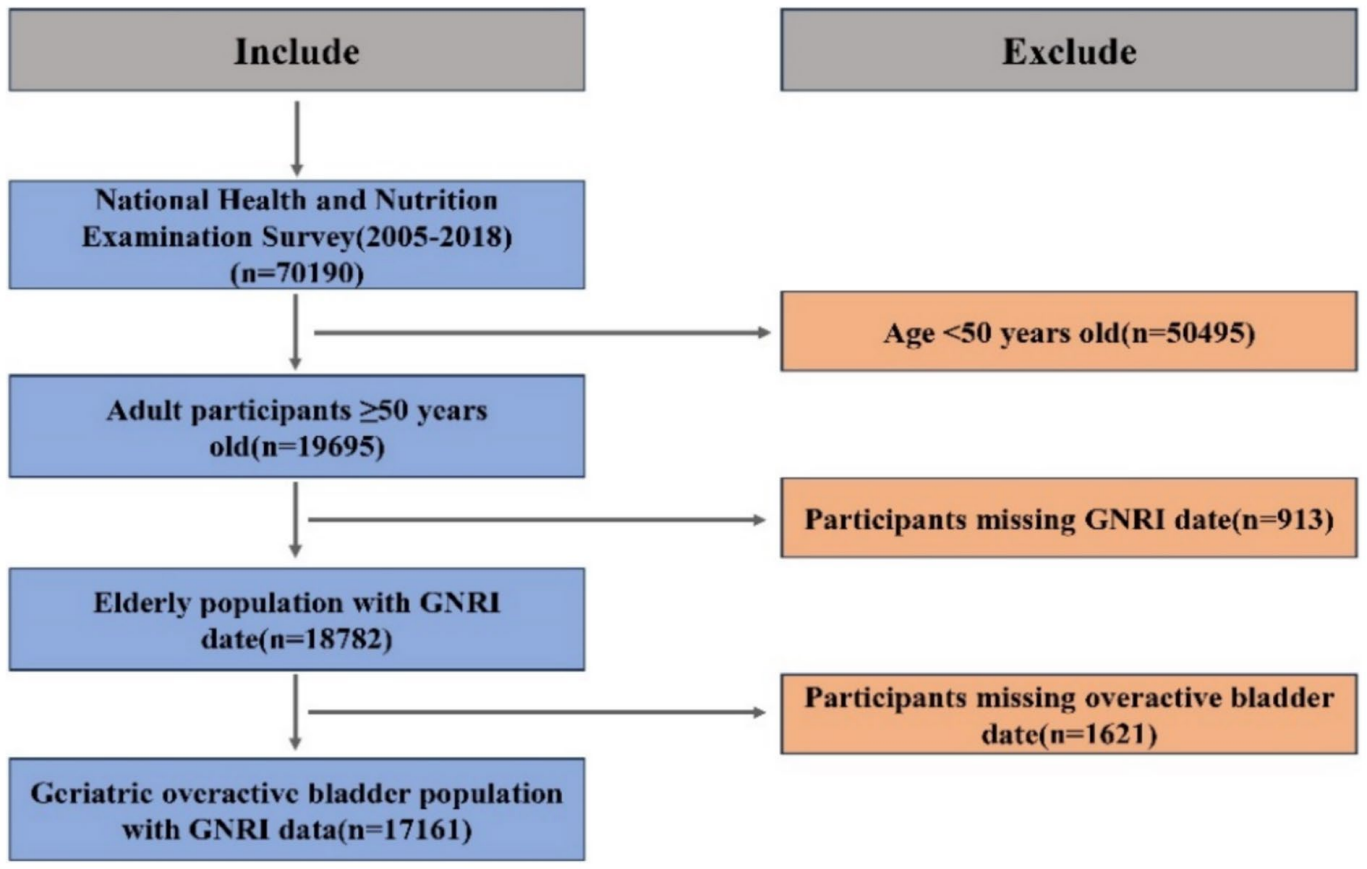
Flowchart of participant selection.

The official NHANES website^1^ served as the primary source of information for this study. NHANES is a comprehensive research initiative conducted by the Centers for Disease Control and Prevention (CDC) in collaboration with the National Center for Health Statistics (NCHS). Its purpose is to provide nationally representative statistics regarding the civilian non-institutionalized population in the United States. All participants provided informed consent prior to interviews and testing, and the NHCS Ethics Review Board approved the NHANES data collection procedures. In this survey, a dataset was created by extracting responses from the publicly accessible NHANES data files between 2005 and 2018. A total of 70,190 individuals participated in two consecutive NHANES survey cycles. Given that OAB is primarily an age-related condition, we excluded individuals under the age of 50 (*n* = 50,495), focusing specifically on the elderly population who are at a higher risk of developing OAB. Epidemiological studies consistently indicate that the prevalence of OAB significantly increases after the age of 50 (16). Subsequently, individuals without calculated GNRI data (*n* = 913) and those missing OAB symptom data (*n* = 1,621) were also excluded. To address potential issues associated with missing data in multivariable analysis, we employed multiple imputation techniques to handle missing values of covariates. The final study population comprised 17,161 individuals. Figure 1 displays a flowchart detailing the criteria used for patient selection.

### GNRI evaluation and grouping

According to the Lorenz formula, the ideal body weight for males is calculated using the formula: height − 100 − [(height − 150)/4]. The specific formula for calculating the GNRI is: GNRI = [41.7 × present body weight (Kg) / ideal body weight (Kg)] + [1.489 serum albumin(g/L)] (17).

If the ratio of current body weight to ideal body weight is one or greater, it is adjusted to equal one for the calculation. Previous studies have calculated the GNRI cut-off values using the critical values of serum albumin and weight loss, thereby determining the GNRI nutritional assessment levels: high nutritional risk (GNRI <98) and low nutritional risk (GNRI ≥98) (11). Patients included in this study were divided into two groups: the high GNRI group (GNRI ≥98) and the low GNRI group (GNRI <98) (18).

### Definition of overactive bladder

According to the definition of OAB, when patients experience urgent urinary incontinence and nocturia, the presence of OAB should be considered (19). At the same time, other potential causes of urination symptoms need to be considered, such as urinary tract infections, prostate diseases, and gynecological diseases. The NHANES database utilizes the Kidney Conditions-Urology questionnaire to assess OAB-related symptoms. Specifically, participants are asked the questions: “During the past 12 months, have you leaked or lost control of your urine, even just a little, because you felt the sudden urge or pressure to urinate and could not get to the toilet in time?” and “How often does this happen?” These two questions evaluate the severity of urgent urinary incontinence (UUI) (20). The severity of nocturia is assessed by the question: “During the past 30 days, how many times do you typically get up to urinate from the time you go to bed at night until you wake up in the morning?” To reinforce the diagnosis of OAB, we used the symptom frequency conversion criteria recorded in NHANES and OABSS scoring to further quantify the symptoms of overactive bladder, with specific scoring criteria shown in Table 1. Finally, the nocturia score and the urgency urinary incontinence score are added together to obtain the overall OABSS score for each participant (21). Individuals with a total score of ≥3 points are considered to be diagnosed with overactive bladder.

**Table 1:** Criteria for conversion of symptom frequencies recorded in NHANES and OABSS scores.

According to NHANES score	According to OABSS score
Urge urinary incontinence frequency	Urge urinary incontinence score
Never	0
Less than once a month	1
A few times a month	1
A few times a week	2
Every day or night	3
Nocturia frequency nocturia score	Nocturia frequency nocturia score
0	0
1	1
2	2
3	3
4	3
5 or more	3

### Covariates

Interviewers determined participants’ date of birth and other demographic data through an initial screening questionnaire, reported by the participants themselves. Age, reported in years, is a continuous variable. Gender is reported as male or female. Race is categorized into Mexican Americans, Other Hispanics, Non-Hispanic White, Non-Hispanic Black, or Other. Body Mass Index (BMI) is calculated as follows: BMI = weight(kg) / height(m^2). Educational attainment is assessed through the initial screening questionnaire and is categorized into Less than high school, High school or equivalent, and College or above. Marital status is divided into Married/Living with Partner, never married, Widowed/Divorced/Separated. Household income is stratified based on the poverty income ratio (22) into three categories: PIR < 1.3, 1.3 ≤ PIR < 3.5, and PIR ≥ 3.5 (23). Smoking status is determined by answering the questions: “Have you smoked at least 100 cigarettes in your entire life?” and “Do you now smoke cigarettes?” Classification of an individual’s smoking status is based on the duration and amount of smoking throughout their lifetime. There are three distinct categories of smoking populations: “Never,” “Former,” and “Current” smokers. The study divides participants into two groups based on whether they engage in excessive alcohol consumption daily. Hypertension is diagnosed based on four criteria, and individuals meeting any of these criteria are classified as hypertensive. The diagnostic criteria include self-reported history of hypertension, use of antihypertensive medication, systolic blood pressure (SBP) ≥ 140 mmHg, or diastolic blood pressure (DBP) ≥ 90 mmHg (24). Participants meeting either of the following two criteria are defined as having diabetes. The diagnostic criteria for diabetes are: the subject uses antidiabetic medication; the patient has a self-reported diagnosis of diabetes (25).

### Statistical analysis

This study utilized Mobile Examination Center sample weights and associated home examination sample design variables to create weights. These weights were employed to ensure representative results consistent with the demographic characteristics of the entire U.S. population. All analyses in this study were conducted with appropriate weighting of the samples according to the NHANES Analytic Guidelines. Continuous variables were reported as weighted means with standard errors, and categorical variables were reported as numerical proportions. Weighted *t*-tests for continuous variables and weighted chi-square tests for categorical variables were used to assess group differences. After adjusting for potential demographic confounding factors, weighted multivariate logistic regression models analyzed the independent association between high GNRI and OAB risk. Subgroup analyses were stratified by age, gender, race, BMI, marital status, education level, poverty rate, smoking, alcohol consumption, and related diseases to examine the correlation of GNRI in different subgroups. To further evaluate the predictive performance of GNRI and other indicators (BMI and serum albumin), we utilized receiver operating characteristic (ROC) curves and the area under the curve (AUC) values. Additionally, causal mediation analysis was conducted to explore the roles of different mediators in this context, estimating the proportions of direct and indirect effects in the association between GNRI and OAB risk. Through causal mediation analysis, the relative contributions of inflammatory cell clusters and cognitive function to the interaction between GNRI and OAB were assessed. Statistical tests were two-tailed, with *p* < 0.05 considered statistically significant. All analyses were performed using R software version 4.4.1 and Free Statistics software version 1.8.

## Results

### Baseline characteristics

Table 1 presents the symptom scoring criteria for participants, who were divided into two groups based on the diagnosis of OAB. A total of 17,161 participants were screened for inclusion in this study. In the preliminary analysis, age (*p* < 0.0001) was identified as a significant factor affecting OAB (Table 2). The mean age of participants with OAB was higher than that of those without OAB, indicating a higher risk of disease in the elderly population. Additionally, there was a significant intergroup difference between females and males (59.85% vs. 40.15%), suggesting a higher risk of disease in the elderly female population. The data indicated a higher proportion of individuals with lower education levels in the OAB group, while the non-OAB group was primarily composed of individuals with a college or higher education level. This suggests that educational attainment may be associated with the risk of OAB (*p* < 0.0001). Furthermore, a higher proportion of divorced or widowed individuals was observed in the OAB group compared to the non-OAB group, which had a higher proportion of married individuals (*p* < 0.0001). It is thus hypothesized that changes in marital status may indirectly affect health status.

**Table 2:** Baseline characteristics of the study population.

Variable	Overall	Non-OAB	OAB	*p*-value
Age (years)	63.40 ± 0.13	62.38 ± 0.14	66.35 ± 0.23	**<0.0001**
Sex (%)				**<0.0001**
Female	8,616 (53.08)	5,654 (50.74)	2,962 (59.85)	
Male	8,545 (46.92)	6,140 (49.26)	2,405 (40.15)	
Race (%)				**<0.0001**
Mexican American	2,278 (4.89)	1,523 (4.51)	755 (5.97)	
Non-Hispanic Black	3,750 (9.68)	2,322 (8.22)	1,428 (13.90)	
Non-Hispanic White	8,000 (75.58)	5,701 (77.38)	2,299 (70.39)	
Other Hispanic	1,660 (3.96)	1,135 (3.80)	525 (4.43)	
Other race – including multi-racial	1,473 (5.90)	1,113 (6.09)	360 (5.32)	
Education (%)				**<0.0001**
Less than high school	4,866 (16.77)	2,936 (14.17)	1930 (24.27)	
High school or equivalent	4,079 (24.84)	2,805 (24.32)	1,274 (26.31)	
College or above	8,216 (58.40)	6,053 (61.51)	2,163 (49.41)	
Smoking status (%)				0.17
Never	5,856 (34.39)	3,977 (34.04)	1879 (35.38)	
Former	8,474 (50.03)	5,890 (50.67)	2,584 (48.18)	
Now	2,831 (15.58)	1927 (15.29)	904 (16.44)	
Marital status (%)				**<0.0001**
Never married	10,194 (65.51)	7,409 (68.12)	2,785 (57.99)	
Married/living with partner	1,184 (5.95)	768 (5.78)	416 (6.42)	
Widowed/divorced/separated	5,783 (28.54)	3,617 (26.10)	2,166 (35.60)	
PIR (%)				**<0.0001**
<1.3	4,971 (17.37)	3,027 (14.68)	1944 (25.15)	
1.3–3.5	5,376 (46.17)	4,186 (50.82)	1,190 (32.75)	
>3.5	6,814 (36.46)	4,581 (34.51)	2,233 (42.10)	
Excessive drinking (%)				0.25
No	11,026 (64.60)	7,694 (64.98)	3,332 (63.52)	
Yes	6,135 (35.40)	4,100 (35.02)	2035 (36.48)	
DM (%)				**<0.0001**
No	11,901 (75.79)	8,626 (78.85)	3,275 (66.95)	
Yes	5,260 (24.21)	3,168 (21.15)	2092 (33.05)	
Hypertension (%)				**<0.0001**
No	6,064 (40.29)	4,660 (43.72)	1,404 (30.41)	
Yes	11,097 (59.71)	7,134 (56.28)	3,963 (69.59)	
BMI (kg.m^2^)	29.37 ± 0.09	28.87 ± 0.10	30.79 ± 0.16	**<0.0001**
Serum albumin (g/L)	41.76 ± 0.06	42.00 ± 0.06	41.09 ± 0.09	**<0.0001**
GNRI (%)				**0.002**
Low-GNRI	1,541 (7.03)	972 (6.53)	569 (8.48)	
High-GNRI	15,620 (92.97)	10,822 (93.47)	4,798 (91.52)	

*P < 0.05 indicated that it was significant.*

Regarding economic factors, a higher proportion of individuals with higher poverty income ratios (22) were found in the non-OAB group, while the OAB group was predominantly concentrated in middle and low-income households. Economic hardship may contribute to the occurrence of OAB (*p* < 0.0001). Analysis of disease factors indicated a higher prevalence of hypertension in the OAB group (69.59% vs. 30.41%, *p* < 0.0001) and a lower prevalence of diabetes (33.05% vs. 66.95%, *p* < 0.0001). Analysis of unhealthy habits showed no significant correlation between smoking and alcohol consumption and the occurrence of OAB.

### The relationship between GNRI levels and OAB

The relationship between the GNRI and the prevalence of OAB was explored using multivariate logistic regression analysis (Table 3), with GNRI divided into two groups. Data analysis revealed that, compared to the low nutritional risk index (Low-GNRI), there was a varying degree of negative correlation between high GNRI levels and OAB across the three models. In the unadjusted model, the results showed a significant association between High-GNRI and OAB prevalence. In the adjusted Model I, demographic parameters such as age, gender, race, and BMI were included as adjustment variables. Adjusted Model II included additional adjustments for education, marital status, PIR, smoking, alcohol consumption, diabetes, and hypertension, in addition to age, gender, race, and BMI. The results were consistent with the unadjusted model. The OR values and *p* values indicated that for each one-unit increase in GNRI, the risk of OAB prevalence was reduced by 0.75 times (*p* = 0.002), 0.74 times (*p* = 0.002), and 0.81 times (*p* = 0.02) in the three models, respectively. Therefore, it can be preliminarily inferred that there is a negative correlation between GNRI levels and the occurrence of OAB, and a high nutritional risk index may be a protective factor in preventing the development of OAB.

**Table 3:** Weighted logistic regression analysis on the association between GNRI and overactive bladder.

Exposure	Non-adjusted		Adjust I		Adjust II	
	OR (95% CI)	*p*-value	OR (95% CI)	*p*-value	OR (95% CI)	*p*-value
Continuous GNRI	0.95 (0.94,0.96)	<0.0001	0.98 (0.97,0.99)	<0.0001	0.98 (0.97,0.99)	<0.001
Low GNRI	Ref		Ref		Ref	
High GNRI	0.75 (0.63,0.90)	0.002	0.74 (0.62,0.89)	0.002	0.81 (0.68,0.97)	0.02

Non-adjusted model adjusts for: GRNI.

Adjust I model adjust for: GNRI, age, sex, race, BMI.

Adjust II model adjust for: GNRI, age, sex, Race, BMI, marital status, PIR, education, DM, hypertension, excessive drinking, smoke.

Low GNRI was the control group.

### Nonlinear relationship between GNRI and OAB prevalence in the elderly

**Figure 2:**
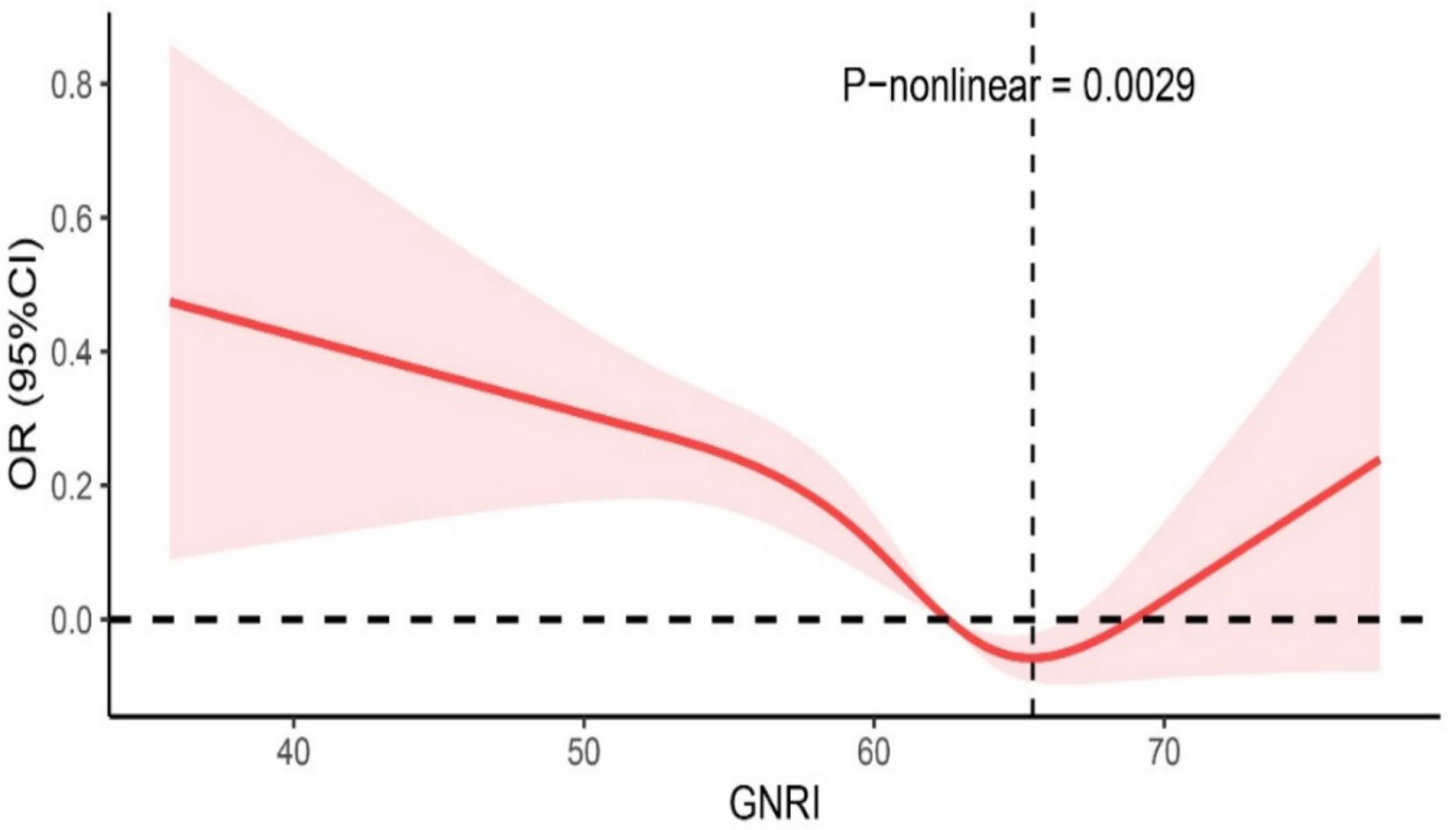
Nonlinear correlation between OAB and GNRI.

The restricted cubic spline regression model demonstrated a non-linear U-shaped association between GNRI and the risk of OAB in the elderly population (P-nonlinear = 0.0029; Figure 2). The negative correlation was limited to when GNRI was less than 63, with a lower risk of OAB in the elderly. However, after the inflection point, the log odds ratio (log OR) of the OAB risk continued to increase with higher levels of GNRI.

### Subgroup analysis

**Figure 3:**
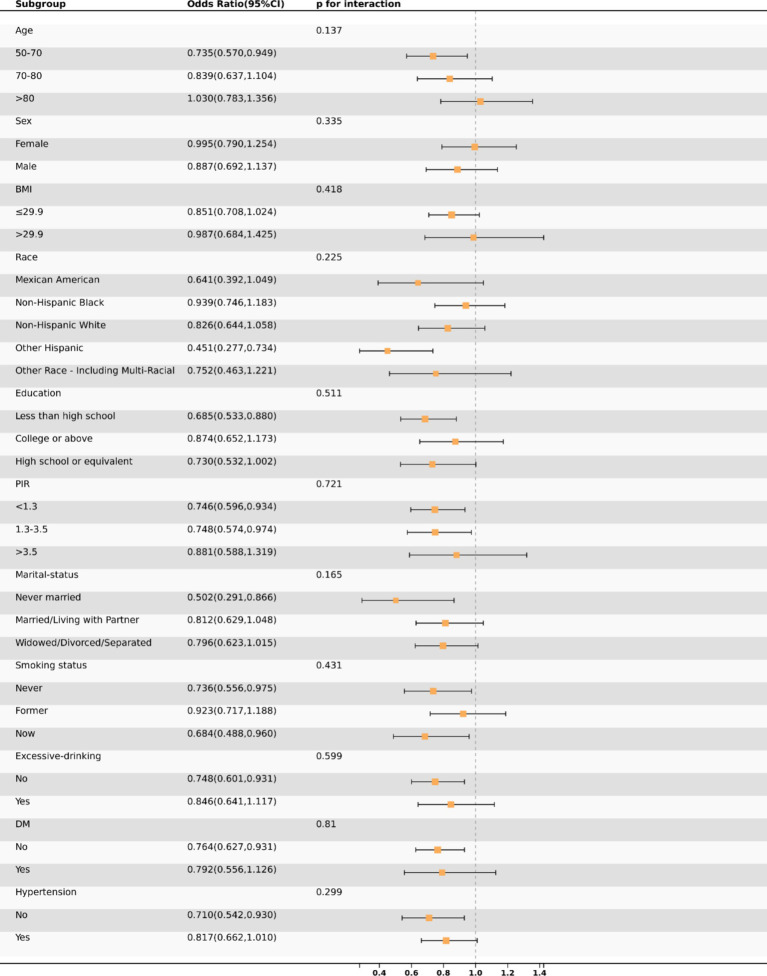
The association between OAB and GNRI in various subgroups.

**Figure 4:**
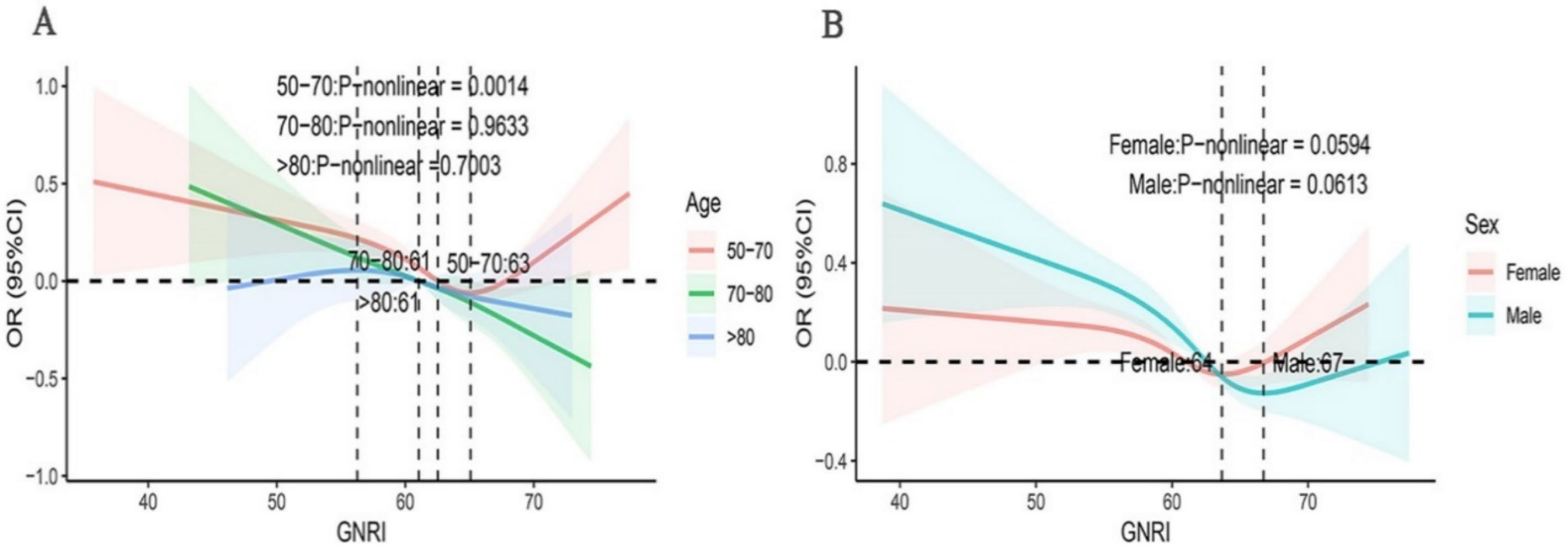
Subgroup analyses using RCS were conducted to examine the association between GNRI and OAB across diverse demographic groups. The analyses were stratified based on gender **(A)**, age **(B)**.

This study employed subgroup analysis to ascertain whether the relationship between OAB and GNRI varied across different subgroups (Figure 3). After adjusting for covariates, we observed no significant differences in the association between OAB and GNRI across subgroups. The relationship between GNRI and the risk of OAB remained consistent across various subgroups, including gender, ethnicity, BMI, PIR, education level, marital status, smoking status, alcohol consumption, hypertension, and diabetes. Specifically, in the elderly population aged 50 to 70 years, the risk of OAB is lower when the GNRI values are below 63. However, beyond this threshold, the risk of OAB increases progressively with higher GNRI values. In contrast, in the population aged 70 to 80 years, GNRI showed a linear negative correlation with the risk of OAB (Figure 4).

### ROC analysis

**Figure 5:**
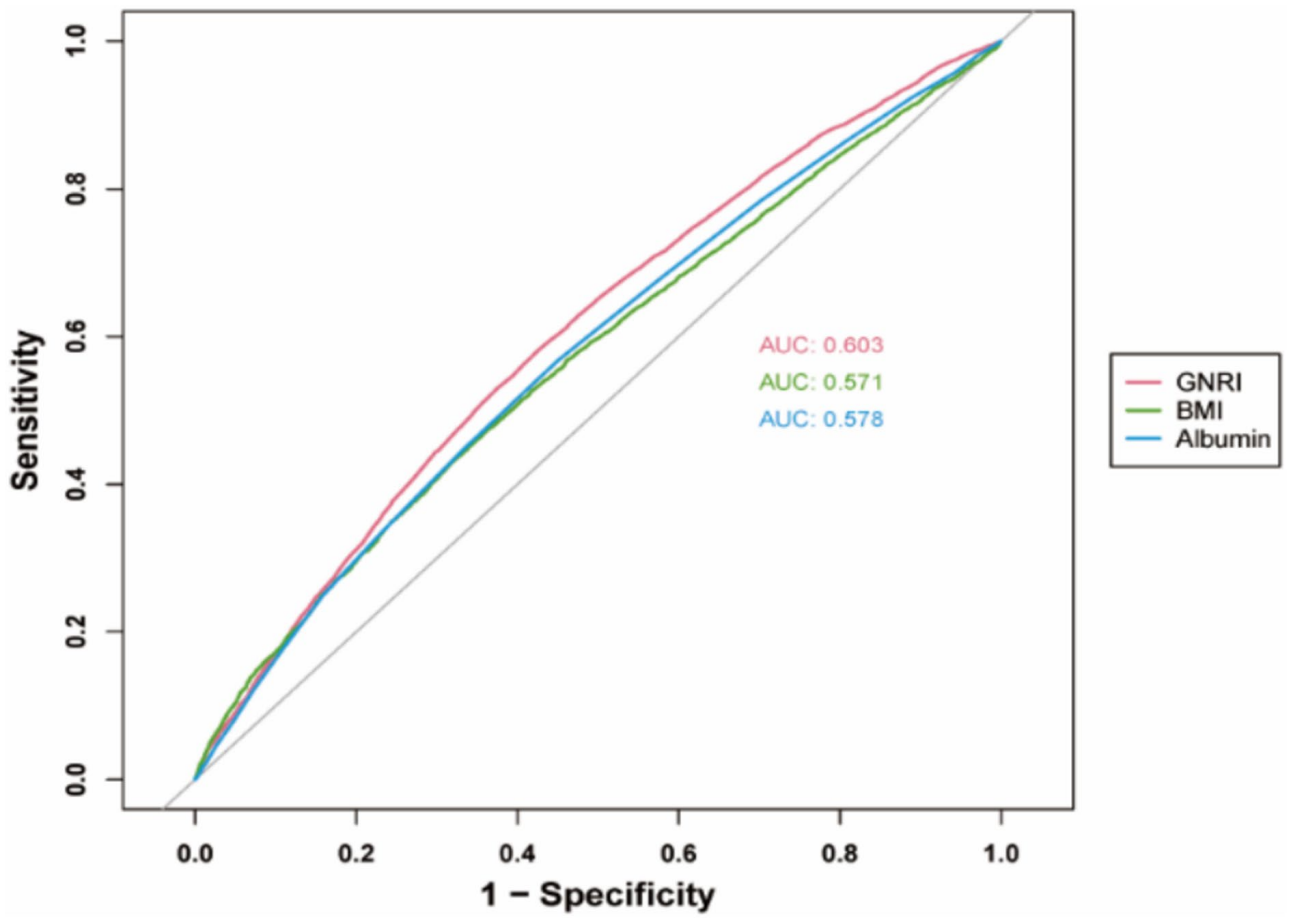
Comparison of diagnostic performance: ROC curves for GNRI, serum albumin, and BMI.

The ROC curves for the predictive abilities of GNRI, serum albumin, and BMI for OAB risk are shown in Figure 5. The AUC for GNRI in the ROC analysis was 0.603, which is higher than that for serum albumin (AUC = 0.578) and BMI (AUC = 0.571). This suggests that, compared to albumin or BMI alone, GNRI may be a more suitable predictor of OAB, although the diagnostic accuracy remains limited.

### Mediation analysis

Among the tested inflammatory markers, the systemic immune inflammation index (SII) exhibited the highest mediation proportion (4.8%), followed by basophils (0.9%), monocytes (0.4%), white blood cells (0.1%), and lymphocytes (0.1%) (Table 4). All inflammatory response markers had a significant overall effect on the association between GNRI and OAB (*p* < 0.001). Cognitive function was measured using the Consortium to Establish a Registry for Alzheimer’s Disease (CERAD), Animal Fluency Test (AFT), and Digit Symbol Substitution Test (DSST) (26, 27). Mediation analysis of cognitive function revealed that the digit symbol score had both direct and indirect effects between GNRI and OAB (*p* < 0.05). The animal fluency score total had only an indirect effect (*p* < 0.001). CERAD confirmed that Alzheimer’s disease had no influence on either.

**Table 4:** Mediation analysis for the associations between GNRI and overactive bladder.

Independent variable	Mediator	Total effect		Indirect effect		Direct effect		Proportion mediated, % (95% CI)
		Coefficient (95% CI)	*p* value	Coefficient (95% CI)	*p* value	Coefficient (95% CI)	*p* value	
GNRI	SII	−0.00229 (−0.00274, −0.00168)	<0.001	−0.00011 (−0.00018, −0.00007)	<0.001	−0.00218 (−0.00264, −0.00157)	<0.001	4.8 (2.9, 8.4)
GNRI	White blood cell count (1,000 cells/uL)	−0.00230 (−0.00275, −0.00170)	<0.001	−0.00000 (−0.00001, 0.00001)	0.480	−0.00230 (−0.00275, −0.00169)	<0.001	0.1 (−0.3, 0.7)
GNRI	Lymphocyte number (1,000 cells/uL)	−0.00229 (−0.00274, −0.00169)	<0.001	−0.00000 (−0.00002, 0.00001)	0.540	−0.00229 (−0.00274, −0.00169)	<0.001	0.1 (−0.3, 0.7)
GNRI	Monocyte number (1,000 cells/uL)	−0.00229 (−0.00274, −0.00170)	<0.001	−0.00001 (−0.00002, 0.00000)	0.140	−0.00228 (−0.00273, −0.00168)	<0.001	0.4 (−0.1, 1.3)
GNRI	Eosinophils number (1,000 cells/uL)	−0.00229 (−0.00274, −0.00169)	<0.001	0.00001 (−0.00001, 0.00004)	0.300	−0.00230 (−0.00276, −0.00171)	<0.001	−0.5 (−2.0, 0.4)
GNRI	Basophils number (1,000 cells/uL)	−0.00229 (−0.00274, −0.00170)	<0.001	−0.00002 (−0.00004, −0.00000)	0.040	−0.00227 (−0.00273, −0.00168)	<0.001	0.9 (0.1, 2.3)
GNRI	Animal fluency score total	0.00058 (−0.00013, 0.00121)	0.160	−0.00007 (−0.00014, −0.00002)	<0.001	0.00065 (−0.00002, 0.00127)	0.120	−8.7 (−94.3, 97.2)
GNRI	Digit symbol score	0.00058 (−0.00015, 0.00119)	0.140	−0.00018 (−0.00029, −0.00010)	<0.001	0.00076 (0.00007, 0.00136)	0.020	−25.0 (−328.0, 183.4)
GNRI	CREAD	0.00061 (−0.00010, 0.00124)	0.140	−0.00006 (−0.00012, 0.00000)	0.060	0.00067 (−0.00001, 0.00130)	0.080	−6.9 (−60.9, 97.1)

The mediation analyses were adjusted for age, sex, race, marital status, PIR, education, smoke, DM and hypertension.

## Discussion

OAB is a complex biological process primarily involving changes in detrusor muscle contraction and neuroregulatory functions of the bladder with age. It is closely associated with numerous physiological or pathological conditions such as age, body weight, smoking, alcohol consumption, diabetes, and neurological disorders. Qin et al. investigated the relationship between muscle mass and lower urinary tract symptoms, with results indicating a positive correlation between the degree of frailty and lower urinary tract symptoms (28). Numerous studies have demonstrated a close association between malnutrition and physical frailty (29, 30). The present study demonstrates a significant correlation between malnutrition and OAB in the elderly population. Subgroup analysis reveals a particularly strong association between the GNRI and the risk of OAB in elderly women. GNRI is an indicator that integrates changes in body weight and levels of serum albumin to assess the nutritional and functional status of elderly individuals (31). A study of 158 patients with OAB showed that low serum albumin levels at admission were associated with the development of OAB (32). In healthy populations, serum albumin is the most abundant protein in plasma, with its concentration accounting for approximately 50% of total plasma protein. Serum albumin plays a crucial role in maintaining vascular oncotic pressure and capillary permeability. A decrease in serum albumin levels may lead to a reduction in plasma oncotic pressure, thereby triggering compensatory diuretic mechanisms to maintain fluid balance and restore oncotic pressure to normal levels (33, 34).

Furthermore, a decrease in serum albumin levels may indicate an impairment in protein synthesis. This synthetic impairment not only affects albumin itself but may also affect the synthesis of other crucial proteins, such as cholinesterase (35). Cholinesterase is a key enzyme that participates in the hydrolysis of acetylcholine. During the activation of the micturition reflex, a decrease in cholinesterase activity may lead to a delayed clearance of acetylcholine released from the parasympathetic nerve terminals of the bladder (36). The accumulation of acetylcholine enhances cholinergic signaling, thereby causing spasms of the detrusor muscle, which may lead to the emergence of symptoms such as urinary frequency and urgency (37, 38).

Existing research has demonstrated a positive correlation between obesity and OAB (39). A recent prospective study conducted by Alsannan et al. found that the risk of OAB in overweight women is 5.8 times higher than in women with a normal BMI, while the risk of severe OAB in obese women is as high as 18.6 times that of women with a normal BMI (40). In this study, the BMI of patients with OAB was significantly higher than that of individuals without OAB. Although the exact mechanisms linking obesity and OAB are not yet fully understood, several possible explanations currently exist. One hypothesis suggests that excess body weight can increase intra-abdominal pressure, thereby causing mechanical compression that affects bladder function (41). Another potential mechanism involves ghrelin. Studies have found that the growth hormone secretagogue receptor (GHSR) is expressed in bladder tissue, particularly in the detrusor muscle. In obese individuals, levels of ghrelin are typically lower, and low levels of ghrelin may upregulate the expression of GHSR in the bladder. This upregulation of expression may alter the function of the detrusor muscle, making it more sensitive to stimuli, and thereby triggering bladder overactivity (42).

There is a potential complex association between inflammation, nutritional status, and OAB. Chronic inflammation can induce changes in bladder function and increased sensitivity, leading to manifestations of OAB cystitis. Inflammatory cytokines (such as interleukin-6 and tumor necrosis factor-*α*) and the release of mast cells lead to inflammatory reactions in bladder tissue (43, 44). On the other hand, inflammation affects nutritional status through various mechanisms, which may in turn promote the development of OAB. Firstly, the inflammatory response can cause anorexia and reduced food intake (45). Pro-inflammatory cytokines act on the appetite regulation centers in the brain, suppressing appetite and leading to insufficient nutrient intake in patients (46). Inflammation and malnutrition can also induce increased muscle catabolism, thereby affecting the function of the detrusor and pelvic floor muscles (47).

This study aimed to analyze data from 17,161 elderly individuals with OAB in the NHANES database from 2005 to 2018. We employed the OABSS questionnaire to assess lower urinary tract symptoms and quantify the severity of bladder overactivity. However, in patients with OAB, the quantitative GNRI score did not reflect the severity of OAB. Using low GNRI as the reference, the binary variable regression analysis between high GNRI and OAB revealed a negative correlation, indicating that high GNRI is a protective factor. This suggests that a lower probability of OAB occurs in individuals with better nutritional status. This finding highlights the significance of nutritional status in the development and management of OAB.

This study provides a reference for the comprehensive assessment and intervention of OAB and emphasizes the importance of health education for the elderly, particularly in terms of nutrition. However, there are several limitations to this study. Firstly, the cross-sectional design of the study does not allow for the determination of a causal relationship between GNRI and OAB. Specifically, it remains uncertain whether a higher GNRI is a decisive factor in the reduced prevalence of OAB or whether OAB itself exacerbates malnutrition. Additionally, the limitations of NHANES data must be considered. NHANES data primarily rely on self-reported information from participants, which may be subject to recall bias, thereby affecting the accuracy of OAB symptoms. The sample selection in NHANES may have certain selection biases, as the voluntary nature of participants may lead to a sample that does not fully represent the characteristics of the entire elderly population. Secondly, although potential confounding factors have been controlled in the study, it is not possible to completely rule out the influence of unidentified residual confounding factors on the results. For example, unincluded chronic diseases (such as chronic kidney disease) or medication use may affect the occurrence and development of OAB. Additionally, psychological factors such as depression and anxiety may also be related to the occurrence of OAB, but these were not fully considered in this study. These omitted confounding factors may lead to biased estimates of the relationship between GNRI and OAB. To mitigate the impact of these potential biases, we employed multivariate regression models in our analysis to control for known confounding factors as much as possible. However, larger prospective studies with greater sample sizes are still needed in the future to explore these potential confounding factors and clarify the causal relationship between GNRI and OAB. Future studies should further investigate the specific relationship between GNRI and OAB symptoms, and more detailed analyses should be conducted on the correlation between GNRI and each symptom of OAB (such as urgency, frequency, and nocturia) to determine whether there are specific nutritional risk factors that affect different symptoms. Additionally, considering the impact of gender and age on OAB, future studies should be conducted separately in males and females, and stratified analyses should be performed in different age groups to reveal potential differences in the relationship between GNRI and OAB. This will help to better understand the role of GNRI in different populations and provide a scientific basis for formulating more personalized nutritional intervention strategies.

## Conclusion

In summary, this study reveals a nonlinear relationship between GNRI and increased OAB risk, particularly in high-risk populations such as elderly female patients. It further establishes the mediating role of inflammatory responses in the GNRI-OAB association. From a clinical perspective, GNRI can be incorporated into routine screening to enhance risk stratification and inform personalized treatment strategies, potentially improving outcomes for OAB patients or those at risk of OAB.

## Data Availability

The original contributions presented in the study are included in the article/supplementary material, further inquiries can be directed to the corresponding authors.

## Footnotes

^1^ https://www.cdc.gov/nchs/nhanes/index.htm
